# Management of Prematurity-Associated Wheeze and Its Association with Atopy

**DOI:** 10.1371/journal.pone.0155695

**Published:** 2016-05-20

**Authors:** Martin O. Edwards, Sarah J. Kotecha, John Lowe, Louise Richards, W. John Watkins, Sailesh Kotecha

**Affiliations:** Department of Child Health, Cardiff University School of Medicine, Cardiff, United Kingdom; University of Southampton, UNITED KINGDOM

## Abstract

**Introduction:**

Although preterm birth is associated with respiratory morbidity in childhood, the role of family history of atopy and whether appropriate treatment has been instituted is unclear. Thus we assessed (i) the prevalence of respiratory symptoms, particularly wheezing, in childhood; (ii) evaluated the role of family history of atopy and mode of delivery, and (iii) documented the drug usage, all in preterm-born children compared to term-born control children.

**Methods:**

We conducted a cross-sectional population-based questionnaire study of 1–10 year-old preterm-born children (n = 13,361) and matched term-born controls (13,361). Data (n = 7,149) was analysed by gestational groups (24–32 weeks, 33–34 weeks, 35–36 weeks and 37–43 weeks) and by age, <5 years old or ≥ 5 years.

**Main Results:**

Preterm born children aged <5 years (n = 2,111, term n = 1,402) had higher rates of wheeze-ever [odds ratio: 2.7 (95% confidence intervals 2.2, 3.3); 1.8 (1.5, 2.2); 1.5 (1.3, 1.8) respectively for the 24–32 weeks, 33–34 weeks, 35–36 weeks groups compared to term]. Similarly for the ≥5 year age group (n = 2,083, term n = 1,456) wheezing increased with increasing prematurity [odds ratios 3.3 (2.7, 4.1), 1.8 (1.5, 2.3) and 1.6 (1.3, 1.9) for the three preterm groups compared to term]. At both age groups, inhaler usage was greater in the lowest preterm group but prematurity-associated wheeze was independent of a family history of atopy.

**Conclusions:**

Increasing prematurity was associated with increased respiratory symptoms, which were independent of a family history of atopy. Use of bronchodilators was also increased in the preterm groups but its efficacy needs careful evaluation.

## Introduction

Preterm birth, defined as birth before 37 weeks’ of gestation, has a major impact on health services across the world, with preterm birth rates varying from 7% in England and Wales to 11.4% in the USA [1, 2]. Children born preterm are known to have a greater prevalence of neurodevelopmental and respiratory problems compared to those born at term [3, 4]. Improved neonatal care has led to greater survival of preterm-born infants, thus the long-term outcomes including respiratory morbidity have become increasingly important [5, 6]. Of note preterm-born children have been shown to be at increased risk of developing wheeze-related disorders [7]; however, the exact nature of the prematurity-associated wheeze has been poorly described, particularly its relationship to atopy and use of inhaler medication. In many studies, prematurity-associated wheezing is labeled as ‘asthma’ but the greater prevalence of respiratory symptoms in preterm-born children than term-born equivalents, suggests possible alternative mechanisms that need identifying. The resulting respiratory morbidity from long-term survival of preterm birth may require a new diagnostic label, which may have altered rates of atopy and different responses to existing inhaled treatments traditionally used in asthma. Some of the outcome data to date has been based on identifying preterm-born subjects from medication use in population-based registries, which has a risk of selection bias and thus potential over diagnosis of asthma and therefore will identify higher rates of treatment [8]. In contrast, other studies report under-treatment of airway obstruction associated with extremes of prematurity [4, 9]. So it is clearly important to assess in a large cohort study the rate of inhaler medication use by children with prematurity-associated wheeze.

Preterm-born children are at risk of developing deficits in percentage forced expiratory volume in one second (%FEV_1_) and have increased admissions to hospital for respiratory related illnesses when compared to term-born children [5, 6, 10]. Both of these studies, Kotecha et al & Paranjothy et al [5, 10], included children born moderate, late and very preterm including those who developed chronic lung disease of prematurity (CLD), who have previously been shown to have the highest risk of developing respiratory morbidity in childhood [11, 12]. In a recent publication using the cohort from the current study, we assessed the independent effects of early term (37 to 38 weeks of gestation), atopy and delivery by caesarian sections in future development of wheezing when compared to full term-born children (39 to 41 weeks of gestation) [13]. However, Astle et al did not show a difference for risk of developing asthma in young children born preterm with CLD compared to children born preterm without CLD [14]. Although Rosas-Salazar et al reported preterm birth was strongly associated with atopic asthma, in a highly selected cohort of Puerto Rican children [15]. Therefore, it is important to establish the characteristics of preterm-born children with and without CLD, who are at increased risk of respiratory symptoms throughout childhood and to what extent these symptoms are related to a family history of atopy and mode of delivery. Furthermore, it is important to establish whether children born preterm with respiratory symptoms are receiving appropriate treatment.

The main study hypothesis was that children born preterm have increased respiratory symptoms; have increased health care utilization and are treated more frequently with inhaler medication when compared to term-born (37 to 42 weeks of gestation) children. Specifically we have quantified the association between gestational age at birth and subsequent respiratory-associated symptoms in preschool (<5 years of age) and school-aged (≥5 years of age) children. We also evaluated the role of a family history of atopy and mode of delivery in the reporting of prematurity-associated wheeze and documented drug usage in preterm-born children classified by gestational groups comparing all the results from the preterm group to the term group.

## Methods

### Study Population

We conducted a cross-sectional population study of children born preterm between 1^st^ January 2003 and 31^st^ December 2011. During this period there were 305,894 live births in Wales of which 22,383 were born preterm (<37 weeks’ gestation). We studied all preterm-survivors born in 2003, 2005 and 2007 thus were aged between 5 and 10 years of age in 2013 (n = 6,406), and those born between 2009 to 2011 thus were aged less than five years of age in 2013 (n = 6,955). In total 13,361 preterm-born children together with an equivalent number of term-born control subjects born on the same day, gender and locality (n = 13,361) were invited to take part. All the subjects were identified from the NHS Welsh Informatics Service (NWIS), which collates data from health databases in Wales including Patient Episode Database for Wales (PEDW, hospital admissions), National Community Child Health Database (NCCHD, birth registration data) and Welsh Demographics Service (WDS, social demographics). The South East Wales Research Ethics Committee approved the study. The parents/guardians of the children, by virtue of completing and returning the questionnaires, consented to participate.

### Survey

Questionnaires were mailed in April 2013 to the parents of the 26,722 subjects with a reminder mailed to non-responders in June 2013. Children aged five years or less were sent the validated Liverpool Respiratory Symptom Questionnaire (LRSQ) [16] and a modified International Study of Asthma and Allergies in Children (ISAAC) questionnaire was mailed to children aged five years or older [9, 17]. The ISAAC questionnaire was modified by including questions relating to respiratory symptoms and the LRSQ was a questionnaire designed to assess respiratory symptoms in young children, having been validated in patients with cystic fibrosis[18]. All returned questionnaires were scanned, validated, checked for accuracy and stored in a secure database (Remark Office OMR 8, Gravic, Philadelphia, USA). Additional data, including gestational age at birth, birth weight, mode of delivery, hospital of birth, the family’s ethnicity and Welsh Index of Multiple Deprivation score (WIMD)[19], a measure of social deprivation based on income, employment, education and health with a range of 1 (most deprived) to 1909 (least deprived), were obtained from NWIS.

### Exposure

The main exposure of interest, gestational age at birth, was based on antenatal ultrasound scans and maternal reporting and recorded in completed weeks of gestation. It was categorized into four groups: 24 to 32 weeks (very preterm), 33 to 34 weeks (moderate preterm), 35 to 36 weeks (late preterm) and 37 to 43 weeks (term controls) as in our previous report [11]. Birth weight was used as a continuous variable and z-scores were calculated using the LMS Growth program (Medical Research Council, UK)[20], which takes into account gestational age and gender. Intrauterine growth restriction (IUGR) was defined as <10^th^ centile for birth weight (after correcting for gender and gestation) and was compared to children with birth weights between 20-80^th^ centile. Participants with birth weights outside ±3.5 SDs or with unknown gestational age were excluded. Of the 7,149 responders, 97 children were excluded due to implausible birth weight or gestational age at birth.

### Outcomes

Respiratory outcomes were based on questionnaire responses and included wheeze-ever, recent wheezing (mild wheeze–a few days per week and severe–almost every day per week), other respiratory symptoms (cough, shortness of breath and/or coryzal symptoms), use of inhalers (bronchodilators and corticosteroids), hospital admissions for chest-related problems in the last twelve months, doctor diagnosed asthma and family history of atopy, which was based on parental reporting of first degree relatives having a history of asthma, eczema and hay fever. Diagnosis of CLD was based on parental reporting and checked with the data on the hospital discharge notification. CLD, which was confined to the children born at ≤32 weeks of gestation as need for supplemental oxygen until at least 36 weeks of corrected gestational age [21, 22]. Parents were also asked if their child had any breathing problems and responded in a free text box. Missing questionnaire responses were recoded as “no” for analysis.

### Statistical analysis

Descriptive analyses were used to summarize the characteristics of all responders, matched case-control responders and of all non-responders. Characteristics included age, gender, gestational age, birth weight, WIMD score, maternal age, maternal smoking during pregnancy, mode of delivery and IUGR. For the characteristics of the responders, we compared results between the gestational groups using appropriate statistical tests (gestational age and birth weight were compared by T-tests; WIMD score by Mann-Whitney U-test; and gender, mode of delivery, IUGR by chi-square) after testing for normality by assessing distribution of frequencies and by performing normal probability plots. Since the response rates were greater amongst the preterm group than the term group, we performed sensitivity analyses by comparing wheezing rates between matched preterm- and term-born children. Because of ethical arrangements, we only had access to identifiable data for responders thus the term-borns were matched with a date of birth within 5 days of the preterm-subjects and with same locality and gender.

The prevalence rates were calculated as a percentage of the study population for each gestational group. The prevalence rates of respiratory symptoms, inhaler treatment, and hospital admissions are reported according to gestational groups with odds ratio to measure the effect size between groups. Separate univariate analyses by ANOVA were constructed for children less than five years-of-age and those five years-of-age and older. Further univariate analyses using ANOVA were conducted to identify risk factors that were associated with wheezing; those statistically significant (p<0.05) were included in multivariable logistic regression analysis for wheezing. Two generalized linear models of multivariable analyses were performed: the first included all significant risk factors and the second included only family history of atopy. Confounding and significant risk factors included in the generalized linear modeling were gender, family history (FH) of atopy, maternal smoking during pregnancy, mode of delivery, maternal history of asthma, breastfeeding at birth, current maternal smoking, social status, ethnicity, and other family member smoking. Adjusted odds ratios were calculated.

A sub-group analysis of wheezers only was performed, by comparing preterm- and term-born wheezers. Univariate analyses using ANOVA were conducted to identify risk factors associated with prematurity-associated wheezing. A separate univariate analysis by ANOVA was performed to assess the influence of family history of atopy on wheezing preterm-born children receiving inhaler treatment compared to wheezing term-born children on inhaler treatment. Analyses were also performed to assess the differences in prevalence of wheezing and inhaler treatment between preterm-born children with and without a family history of atopy and term-born children with and without a family history of atopy. Finally a sub-group analysis of the very preterm group was performed to assess the differences in prevalence of wheezing and inhaler treatment between children with CLD and those without CLD. The statistical analyses were performed using PAWS (version 18.0, SPSS Inc, Chicago, IL; US). p<0.05 was considered significant.

## Results

### Participant characteristics

**Fig 1:**
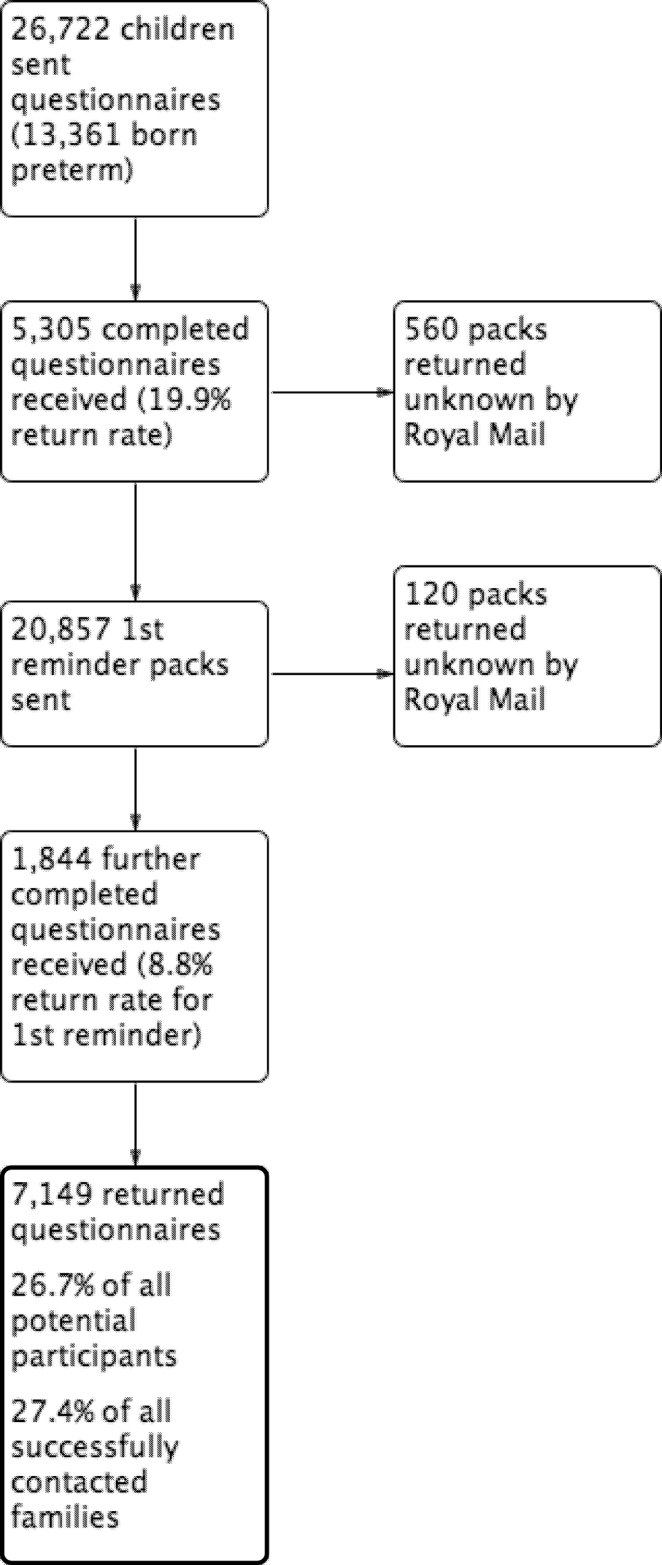
Diagram showing return rates of completed questionnaires in the Respiratory and Neurological Outcomes of children born Preterm Study (RANOPS).

There were 7,149 (26.7%) responses with 4,284 (60%) from preterm-born and 2,865 (40%) from term-born children (Fig 1). The characteristics of participants are shown in Tables 1 and 2 and comparisons to non-responders are shown in Tables A and B in S1 File. When comparing the different characteristics of the descriptive analysis of included preterm-born children to term-born controls using appropriate parametric or non-parametric tests, there were no significant differences for gender, age of child or maternal age at time of delivery. However, a trend was noted for higher antenatal maternal smoking rates, greater delivery rates by caesarean section and lower WIMD ranks in the preterm-born groups when compared to the term group. There were 1,260 pairs of matched preterm- and term-born responders (total 2,520). The characteristics were similar between the matched responders and all responders (Tables F and G in S1 File).

**Table 1:** Characteristics of responders <5 years of age according to gestational groups.

	Very Preterm	Moderate Preterm	Late Preterm	Full Term
	N = 519	N = 486	N = 1,149	N = 1,403
**Gestational age, weeks**	29.7*	33.7*	35.7*	39.6*
** (mean, 95%CI)**	(29.5,29.9)	(33.6,33.7)	(35.6,35.7)	(39.6,39.7)
**Birthweight, kg**	1.45*	2.18*	2.64*	3.46*
** (mean, 95%CI)**	(1.41, 1.50)	(2.14, 2.22)	(2.61, 2.66)	(3.43, 3.49)
**Male (%)**	260 (50%)	275 (57%)	621 (54%)	743(53%)
**WIMD rank (median) Range: 1–1909**	898	929	934	997
**Age, years**	2.24	2.22	2.29	2.24
** (mean, 95% CI)**	(2.16, 2.32)	(2.14, 2.30)	(2.24, 2.35)	(2.19, 2.28)
**Antenatal maternal smoking**	72/511 (14.1%)	56/483 (11.6%)	136/1,143 (11.9%)	133/1,385 (9.6%)
**Maternal age, years**	30.5	30.5	30.1	30.3
** (mean, 95%CI)**	(30.0, 31.1)	(30.0, 31.0)	(29.7, 30.5)	(30.0, 30.6)
**Mode of delivery (CS or not)**	288 (55.5%)*	263 (54.1%)*	505 (44%)*	378 (26.9%)*
**Missing data**	33 (6.4%)	23 (4.7%)	60 (5.2%)	46 (3.3%)
**IUGR**	71(13.7%)*	64(13.2%)*	111(9.7%)*	97(6.9%)*

*Significant difference (p<0.001) between groups.

**Table 2:** Characteristics of responders’ ≥5 years of age according to gestational groups.

	Very Preterm	Moderate Preterm	Late Preterm	Full Term
	N = 509	N = 466	N = 1,155	N = 1,462
**Gestational age, weeks**	29.8*	33.6*	35.6*	39.6*
** (mean, 95%CI)**	(29.6, 30.0)	(33.6, 33.7)	(35.6, 35.7)	(39.6, 39.7)
**Birthweight, kg**	1.45*	2.22*	2.64*	3.46*
** (mean, 95%CI)**	(1.40, 1.49)	(2.18, 2.26)	(2.61, 2.67)	(3.44, 3.49)
**Male (%)**	301 (59.1%)	260 (55.8%)	627 (54.2%)	775 (53%)
**WIMD rank (median) Range: 1–1909**	997*	936*	1,019*	1,110*
**Age, years**	7.1	7.1	7.3	7.2
** (mean, 95%CI)**	(7.0, 7.3)	(7.0, 7.3)	(7.2, 7.4)	(7.1, 7.3)
**Antenatal maternal smoking**	76/475 (16%)	67/432 (15.5%)	175/1,080 (16.2%)	184/1,343 (13.7%)
**Maternal age, years**	29.7	30.4	30.1	30.4
** (mean, 95%CI)**	(29.2, 30.4)	(29.8, 31.0)	(29.7, 30.4)	(30.1, 30.7)
**Mode of delivery (CS or not)**	269 (52.8%)*	229 (49.1%)*	439 (38%)*	344 (23.5%)*
**Missing data**	53 (10.4%)	41 (8.8%)	76 (6.6%)	162 (11.1%)
**IUGR**	77 (15.2%)*	39 (8.4%)*	116 (10.1%)*	95 (6.5%)*

*Significant difference (p<0.001) between groups.

### Risk factors

Lower gestational age at birth, male gender, maternal smoking during pregnancy, current maternal smoking, other family member current smoking, maternal history of asthma, delivery by caesarean section (CS), family history of atopy, lower WIMD rank, and ethnicity were all, individually, significantly associated with increased rates of wheezing for both age groups (p <0.05). These risk factors were all included as in the multivariable analysis of wheezing symptoms (Tables 3 and 4). Influence of CLD was analysed separately as it was confined to the very preterm group (see below).

**Table 3:** Childhood wheezing, family history, inhaler use and hospital admissions for all children less than 5 years-of-age compared by gestational age (unadjusted OR).

	Very Preterm	Moderate Preterm	Late Preterm	Full Term
	N = 502	N = 479	N = 1,130	N = 1,402
**Wheeze-ever (%)**	325 (64.7%)	266 (55.5%)	581 (51.4%)	571 (40.7%)
**OR (95% CI)**	2.7 (2.2, 3.3)	1.8 (1.5, 2.2)	1.5 (1.3, 1.8)	
**p-value**	<0.001	<0.001	<0.001	
**aOR (95% CI)**^**#**^	2.6 (2.1, 3.3)	1.8 (1.4, 2.2)	1.5 (1.2, 1.7)	
**aOR (95% CI)^**	2.8 (2.2, 3.4)	1.9 (1.5, 2.3)	1.5 (1.3, 1.8)	
**Recent wheeze (%)**	201 (40%)	160 (33.4%)	326 (28.8%)	261 (18.6%)
**OR (95% CI)**	2.9 (2.3, 5.7)	2.2 (1.7, 2.8)	1.8 (1.5, 2.1)	
**p-value**	<0.001	<0.001	<0.001	
**aOR (95% CI)**^**#**^	2.9 (2.3, 3.6)	2.2 (1.7, 2.8)	1.7 (1.4,2.0)	
**aOR (95% CI)^**	3.0 (2.4, 3.8)	2.3 (1.8, 2.9)	1.7 (1.4,2.1)	
**Mild wheeze (%)**	183 (36.5%)	146 (30.5%)	291 (25.8%)	238 (17%)
**OR (95% CI)**	2.8 (2.2, 3.5)	2.1 (1.7, 2.7)	1.7 (1.4, 2.1)	
**p-value**	<0.001	<0.001	<0.001	
**Severe wheeze (%)**	18 (3.6%)	14 (2.9%)	35 (3.1%)	23 (1.6%)
**OR (95% CI)**	2.2 (1.2, 4.2)	1.8 (0.9, 3.5)	1.9 (1.1, 3.3)	
**p-value**	<0.05	0.1	<0.05	
**Family history of atopy (%)**	131 (26.1%)	115 (24%)	355 (31.4%)	380 (27.1%)
**OR (95% CI)**	1.0 (0.8,1.2)	0.9 (0.7, 1.1)	1.2 (1.0, 1.5)	
**p-value**	0.662	0.184	0.018	
**Any inhaler medication use (%)**	167 (33.3%)	95 (19.8%)	222 (19.6%)	167 (11.9%)
**OR (95% CI)**	3.7 (2.9, 4.7)	1.8 (1.4, 2.4)	1.8 (1.5, 2.5)	
**p-value**	<0.001	<0.001	<0.001	
**Corticosteroid inhaler medication use (%)**	52 (10.4%)	27 (5.6%)	76 (6.7%)	41 (2.9%)
**OR (95% CI)**	3.8 (2.5, 5.9)	2.0 (1.2, 3.3)	2.4 (1.6, 3.5)	
**p-value**	<0.001	0.007	<0.001	
**Hospital admission with breathing related problem (last 12 months) (%)**	110 (21.9%)	58 (12.1%)	112 (9.9%)	77 (5.5%)
**OR (95% CI)**	4.8 (3.5, 6.6)	2.4 (1.7, 3.4)	1.9 (1.4, 2.6)	
**p-value**	<0.001	<0.001	<0.001	

^**#**^Confounding factors–gender, FH of atopy, maternal smoking during pregnancy, CS, maternal history of asthma, breastfeeding at birth, current maternal smoking, social status, ethnicity, and other family member smoking.

^^^adjusted for Family history of atopy only.

**Table 4:** Childhood wheezing, family history, inhaler use and hospital admissions for all children 5 years-of-age and older compared by gestational age (unadjusted OR).

	Very Preterm	Moderate Preterm	Late Preterm	Full Term
	N = 495	N = 450	N = 1,138	N = 1,456
**Wheeze-ever (%)**	276 (55.8%)	186 (41.3%)	430 (37.8%)	403 (27.7%)
**OR (95% CI)**	3.3 (2.7, 4.1)	1.8 (1.5, 2.3)	1.6 (1.3, 1.9)	
**p-value**	<0.001	<0.001	<0.001	
**aOR (95% CI)**^**#**^	3.3 (2.7, 4.2)	1.8 (1.4, 2.2)	1.5 (1.3, 1.8)	
**aOR (95% CI)^**	3.5(2.8, 4.3)	1.9 (1.5, 2.4)	1.6 (1.3, 1.9)	
**Recent wheeze (%)**	146 (29.5%)	92 (20.4%)	227 (19.9%)	217 (14.9%)
**OR (95% CI)**	2.4 (1.9, 3.0)	1.5 (1.1, 1.9)	1.4 (1.2, 1.7)	
**p-value**	<0.001	0.006	0.001	
**aOR (95% CI)**^**#**^	2.4 (1.9, 3.1)	1.4 (1.0, 1.8)	1.3 (1.1, 1.7)	
**aOR (95% CI)^**	2.6 (2.0, 3.3)	1.5 (1.1, 2.0)	1.4 (1.1, 1.7)	
**Doctor diagnosis of asthma (%)**	127 (25.7%)	78 (17.3%)	197 (17.3%)	182 (12.5%)
**OR (95% CI)**	2.4 (1.9, 3.1)	1.5 (1.1, 2.0)	1.5 (1.2, 1.8)	
**p-value**	<0.001	<0.01	<0.001	
**Family history of atopy (%)**	91 (18.4%)	98 (21.8%)	275 (24.2%)	330 (22.7%)
**OR (95% CI)**	0.8 (0.6, 0.99)	1.0 (0.7, 1.2)	1.1 (0.9, 1.3)	
**p-value**	0.046	0.694	0.370	
**Any inhaler medication use (%)**	109 (22%)	66 (14.7%)	162 (14.2%)	159 (10.9%)
**OR (95% CI)**	2.3 (1.8, 3.0)	1.4 (1.0, 1.9)	1.4 (1.1, 1.7)	
**p-value**	<0.001	0.032	0.011	
**Corticosteroid inhaler medication use (%)**	66 (13.3%)	36 (8%)	92 (8.1%)	92 (6.3%)
**OR (95% CI)**	2.3 (1.6, 3.2)	1.3 (0.9, 1.9)	1.3 (0.97, 1.8)	
**p-value**	<0.001	0.21	0.08	
**Hospital admission with breathing related problem (last 12 months) (%)**	17 (3.4%)	16 (3.6%)	24 (2.1%)	23 (1.6%)
**OR (95% CI)**	2.2 (1.2, 4.2)	2.3 (1.2, 4.4)	1.3 (0.7, 2.3)	
**p-value**	0.014	0.012	0.318	

^**#**^Confounding factors–gender, FH of atopy, maternal smoking during pregnancy, CS, maternal history of asthma, breastfeeding at birth, current maternal smoking, social status, ethnicity, and other family member smoking.

^^^adjusted for Family history of atopy only.

### Prevalence

The prevalence of wheeze-ever in children less than five years-of-age was 64.7%, 55.5%, 51.4% and 40.7% in the very preterm, moderate, late preterm and term groups respectively with odds ratios of 2.67 (95%CI 2.2, 3.3), 1.82 (95%CI 1.5, 2.2) and 1.54 (95%CI 1.3, 1.8) respectively (Table 3). Slightly higher odds ratios were noted for recent wheeze (over the last three months), severity of wheezing, use of inhaler medication including corticosteroids and admission to hospital over the last 12 months for a respiratory illness. Family history of atopy showed a prevalence of 26.1% in the very preterm group, 24% in the moderate preterm group, 31.4% in the late preterm group and 27.1% in the term controls; only the late preterm group was significantly different to the term controls (p = 0.018). Multivariable logistic regression analyses (a) including all significant risk factors described above, and (b) including only family history of atopy, showed results essentially the same as for univariate analysis for association of prematurity and subsequent wheezing at both age groups.

The prevalence of wheeze-ever in children aged five years or older was 55.8%, 41.3%, 37.8% and 27.7% respectively for the very preterm, moderate, late preterm and term groups with odds ratios of 3.29 (95%CI 2.7, 4.1), 1.84 (95%CI 1.5, 2.3) and 1.58 (95%CI 1.3, 1.9) respectively (Table 4). Slightly lower odds ratios were noted for recent wheeze (over the last 12 months), doctor diagnosed asthma, use of inhaler medication including corticosteroids, and admission to hospital over the previous 12 months for a respiratory illness. Family history of atopy showed a prevalence of 18.4%, 21.8%, 24.2% and 22.7% for the very preterm, moderate, late preterm and term groups respectively, with only the very preterm group being marginally different to the term controls (p = 0.046). Multivariable logistic regression analyses (a) including all significant risk factors and (b) including only family history of atopy, showed results essentially the same as for univariate analysis for association of prematurity and subsequent wheezing at both age groups. The wheezing rates of the term-born matched preterm-born responders (both age groups) were similar to the results of all responders (Tables H and I in S1 File).

The results of the sub-group analysis on only wheezing children identified the following significant risk factors for preterm-born wheezy children aged less than five years: being delivered by CS, IUGR, and current maternal smoking (Table C in S1 File). For the group aged five years and older, the significant factors were delivery by CS and IUGR (Table D in S1 File). Rates of family history of atopy were similar in the preterm- and term-born wheezing children on inhaler treatment for both age’s groups (Table 5).

**Table 5:** The influence of family history of atopy on wheezing children being treated with inhalers by comparing preterm to term-born participants for both age groups.

**Wheezing children on inhaler treatment (<5 years old)**	**Preterm**	**Term**	**OR (95% CI)**
**Family history of Atopy +**	187/458 (40.8%)	64/154 (41.6%)	1.0 (0.7, 1.4)
**Family history of Atopy -**	271/458 (59.2%)	90/154 (58.4%)	P = 0.8
**Wheezing children on inhaler treatment (≥5 years old)**	**Preterm**	**Term**	**OR (95% CI)**
**Family history of Atopy +**	116/330 (35.2%)	57/156 (36.5%)	0.9 (0.6, 1.4)
**Family history of Atopy -**	214/330 (64.8%)	99/156 (63.5%)	p = 0.8

**Fig 2:**
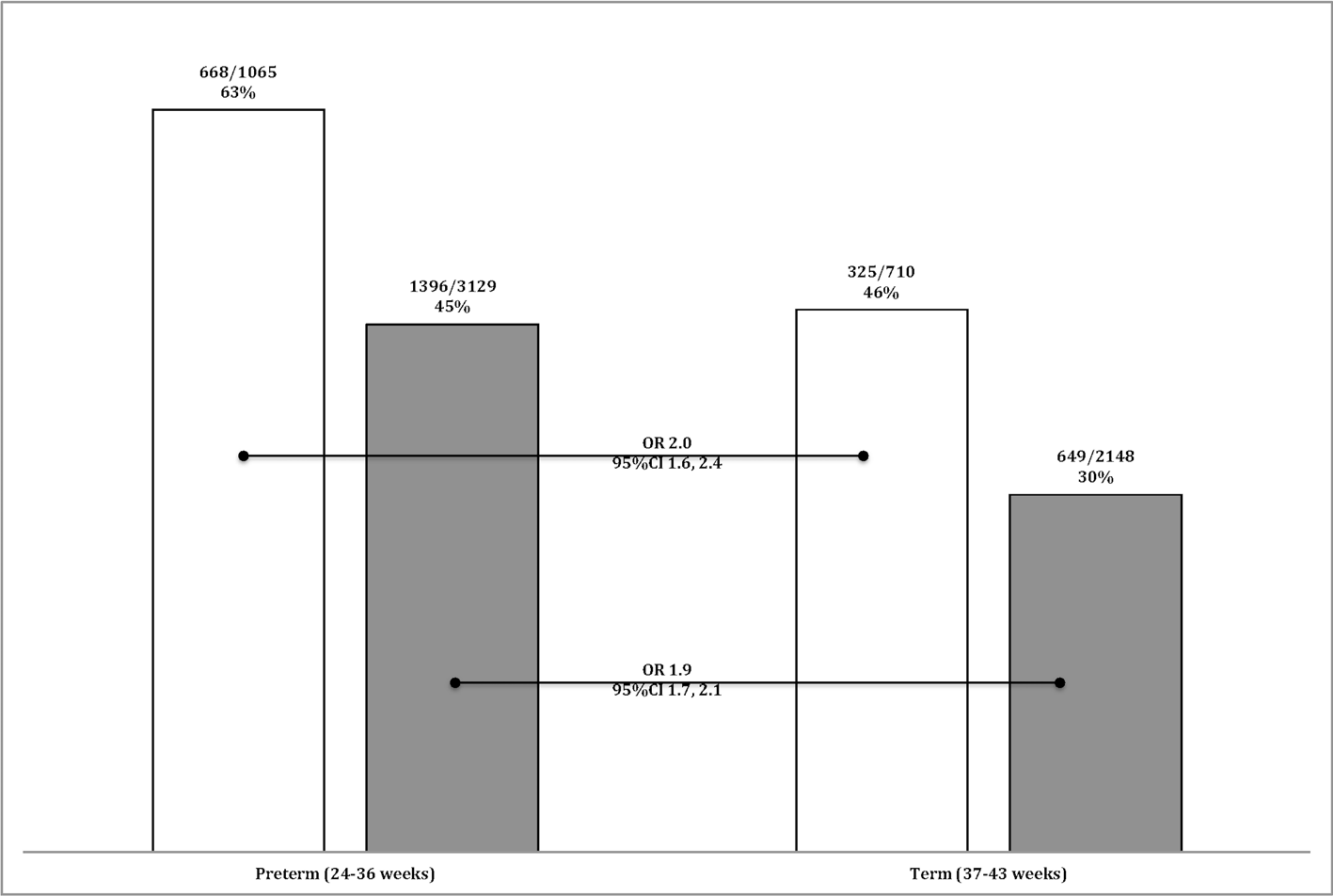
Prevalence of ever wheezing for the whole study population. Bars denote the percentage of children having ever wheezed who have a family history of atopy (white bars) or not (black bars) and for both gestational groups, preterm or term.

**Fig 3:**
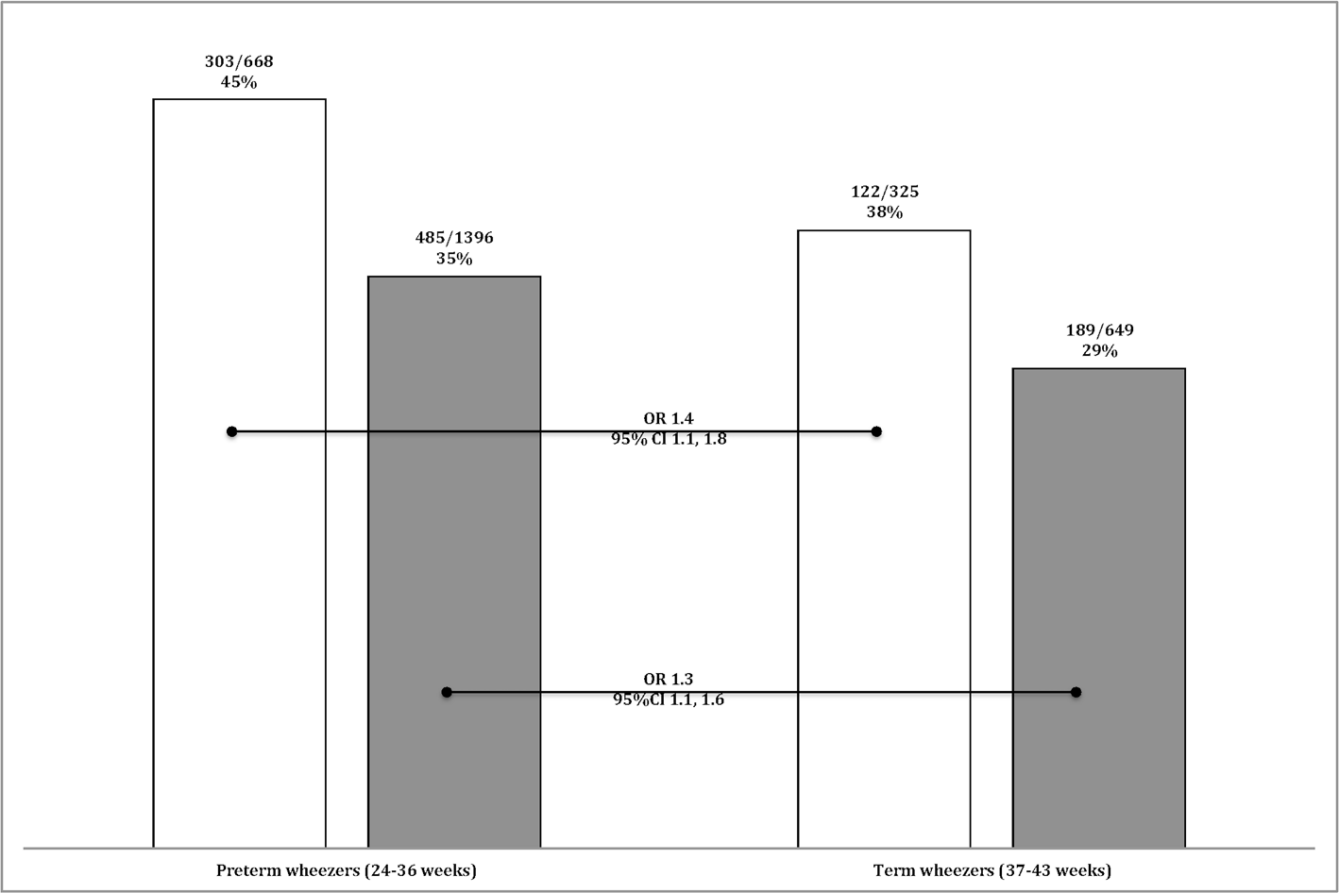
Prevalence of inhaler treatment amongst wheezing children. Bars denote the percentage of wheezy children being treated with inhalers who have a family history of atopy (white bars) or not (black bars) and for both gestational groups, preterm or term.

Preterm-born children who have a family history of atopy, had a greater risk for wheezing than term-born children with a family history of atopy (OR 2.0, 95%CI 1.6, 2.4). However, preterm-born children *without* a family history of atopy also had similar greater risk of wheezing than children born term *without* a family history of atopy (OR 1.9, 95%CI 1.7, 2.1) suggesting that atopy does not play a part in the prematurity-associated wheeze (Fig 2). Similarly, preterm-born children with wheezing and a family history of atopy were treated with inhalers more frequently than term-born children with wheeze and family history of atopy (OR 1.4, 95%CI 1.1, 1.8) which was similar to the comparison between preterm- and term-born children with wheezing and no family history of atopy (OR 1.3, 95%CI 1.1, 1.6) (Fig 3).

Children with CLD, who were confined to the very preterm group, had lower birth weight and gestational age than very preterm without CLD (Table E in S1 File). The CLD group had greater rates of wheezing (71.7%), inhaler use (37.5%) and hospital admissions (17.1%) compared to term-born controls (Table 6). Children with CLD had significantly greater rates of wheezing and inhaler use compared to the very preterm group without CLD. Children who were born very preterm without CLD also had significantly greater rates of wheezing (58.2%), inhaler use (25.6%) and hospital admissions (12%) when compared to term-born controls. Family history of atopy was lower (p<0.05) between children with CLD (17.8%) and term-born controls, and similar between very preterm children without CLD (23.1%) and term-born controls (24.8%).

**Table 6:** Childhood wheezing, family history, inhaler use and hospital admissions for very preterm born children with or without CLD compared to full term (unadjusted OR).

	CLD	No CLD	Full term
	N = 152	N = 845	N = 2,858
**Wheeze-ever (%)**	109 (71.7%)	492 (58.2%)	974 (34.1%)
**OR (95% CI)**	4.9 (3.4, 7.0)	2.7 (2.3, 3.2)	
**p-value**	<0.001	<0.001	
**Family history of atopy (%)**	27 (17.8%)	195 (23.1%)	710 (24.8%)
**OR (95% CI)**	0.65 (0.4, 0.999)	0.9 (0.8, 1.1)	
**p-value**	0.049	N.S.	
**Any inhaler medication use (%)**	57 (37.5%)	219 (25.95%)	326 (11.4%)
**OR (95% CI)**	4.7 (3.3, 6.6)	2.7 (2.2, 3.3)	
**p-value**	<0.001	<0.001	
**Hospital admission with breathing related problem (last 12 months) (%)**	26 (17.1%)	101 (12%)	100 (3.5%)
**OR (95% CI)**	5.7 (3.6, 9.1)	3.7 (2.8, 5.0)	
**p-value**	<0.001	<0.001	

Comparisons are between the very preterm group with CLD and without CLD groups against the term group.

## Discussion

In this cross-sectional population-based cohort study, we explored the respiratory health outcomes including symptoms, family history of atopy and inhaler treatment of children-born preterm. Our results show that children (aged 1–10 years of age) born preterm have greater respiratory symptoms, increased hospital admissions for respiratory related morbidity and greater use of inhaler medication compared to term-born children. A gradient across the gestational age groups was observed with those born very preterm having the highest prevalence rates. Importantly, we show that prematurity-associated wheeze appears to be independent of a family history of atopy. Our results confirm that children born very preterm have significantly higher prevalence of respiratory symptoms including wheezing during pre-school aged years as previously reported [23, 24] but also during early school-aged years when compared to children born full term. Prevalence of wheezing was increased in those with CLD and in those without CLD when compared to the term population. For the whole population of preterm-born children aged less than five years, the overall odds ratio for wheeze-ever was 1.8 (95% CI 1.6, 2.1), which is remarkably similar to those reported previously [7].

Our study also noted that preterm-born children were being treated with inhaler medication (bronchodilators and corticosteroids) with greater frequency than term-born children. This too showed a gradient effect with increasing drug usage with decreasing gestational age at birth. Some studies have previously reported under treatment especially in those born very preterm with CLD [4, 9]. Whether the bronchodilators or corticosteroids are efficacious is speculative and will require further studies. Other studies have identified an increased number of prescriptions for inhaler medication for preterm-born children; however these are registry-based studies that have been restrictive in their study population by identifying subjects by diagnosis of asthma, thus the results are unsurprisingly associated with increased inhaler usage and can not therefore comment on non-asthmatic forms of wheezing in childhood [25, 26]. Our study specifically asked for inhaled drug usage, independently of any diagnostic labels given to the child, thus is likely to be representative of the population of preterm-born children.

Our recent systematic review of studies evaluating efficacy of inhaled bronchodilator identified 21 studies but only one study from 1997, assessed longer-term response to terbutaline showing an improvement [27]. The remaining studies only assessed responses to single doses of inhaled bronchodilators generally showing positive responses [28]. Similarly studies assessing the role of inhaled corticosteroids in this group of children are also limited [29, 30], and both showed no significant improvement following treatment with corticosteroids. Thus treatment needs to be evaluated in well-designed, adequately powered studies if we are to optimally treat prematurity-associated wheezing and avoid untoward adverse effects of steroids, especially growth suppression [31].

A meta-analysis of >147,000 children from 31 European birth cohorts showed that children born a lower gestational age or with accelerated infant weight gain have increased risk of childhood asthma [32]. The authors speculated that adaptation of the immune system in preterm born children maybe a possible cause of this increased risk of asthma. However, they were not able to assess the impact of atopy in prematurity-associated wheeze or treatment of the prematurity-associated wheezing. The increase in reported wheeze and other respiratory symptoms in preterm-born children could potentially be associated with atopy as shown by Rosas-Salaza et al [15], thus we explored if a family history of atopy was associated with the increased reported respiratory symptoms in our population. The prevalence was largely the same between the preterm and term groups at both ages. In addition, although the rates of wheezing were increased in preterm-born subjects with a family history, there was a similar increase in respiratory symptoms in subjects without a family history of atopy. Family history of atopy was not significantly associated with respiratory symptoms when included in regression models. Thus the prematurity-associated wheeze appears not to be associated with a family history of atopy.

In our study, the risk factors most strongly associated with wheeze-ever in preterm-born children were the degree of prematurity with decreasing gestation associated with increasing symptoms; mode of delivery and IUGR for both age groups; and current maternal smoking for the less than 5-years of age group. Mode of delivery is likely to be associated with the degree of prematurity but IUGR has been reported to be associated with increased respiratory disease in preterm-born infants in the neonatal period [33] and in term-born children [34].

The mechanisms underlying wheezing disorders following preterm birth are unclear. Children with atopic asthma have obstructive airflow secondary to airway inflammation and bronchial hyper-responsiveness, which is reversible with bronchodilators. However, preterm-born children, or even those born early-term[13], with respiratory symptoms are likely to have alternative mechanisms, which may not respond similarly to current inhaled therapies. Prematurity is clearly associated with delivery at an early stage of lung growth and subsequent lung development may be disturbed [35]. Some studies have shown that maternal antibiotic use during pregnancy increases the risk of asthma in early childhood suggesting a disturbance in the bacterial ecology of the foetus may lead to asthma [36]. This may only be in relation to asthma and, as prematurity-associated wheezing may have a different phenotype, different mechanisms may be involved [37, 38]. Other possible mechanisms include early exposure to respiratory viral infections, accelerated postnatal growth and swallowing dysfunction leading to recurrent aspiration [32, 39, 40].

In survivors of CLD, the underlying lung pathology may resemble that of pulmonary emphysema rather than asthma [41]. Our data shows that those with CLD had a greater rate of wheezing when compared to those without CLD, as confirmed by our recent systematic review[5]. Most studies, but without any adequate randomised control trials, have focused on responses to individual doses of bronchodilators especially on children who had CLD in infancy. Exhaled nitric oxide studies on children surviving CLD have not consistently shown any increases in symptomatic children suggesting that eosinophilic dominant disease is unlikely to explain the wheezing in preterm children [33]. Interestingly, individual reports suggest neutrophilic or oxidant injury may be continuing [42, 43]. However these are small studies that need confirming and, more importantly, if these are primary or secondary effects of prematurity and if they are modified by regular use of inhaled drugs.

### Study Limitations

Our study has a few limitations. It is a cross-sectional study relying on parental reporting of symptoms. Thus there is potential for recall bias. This may have affected the wheezing rates for children over 5 years of age, which reports a lower rate of ever wheezing than children less than 5 years of age. However previous studies suggest that parental recall of acute illness correlates well with medical records [44, 45]. We also included additional information from health database to provide more comprehensive assessment and confirm diagnoses such as CLD from doctor diagnosis. Ascertainment bias may have overestimated our results, as preterm-born children are known to present more frequently for healthcare reviews thus may result in greater diagnosis and possibly subsequent inhaler medication. However this is likely to be applicable to both preterm and term-born groups and so unlikely to affect the overall results. There was also a low response rate at 26.7%, even though previous studies had shown a good response from families in Wales [46]. This may have resulted in recruitment bias and as shown by our comparison between responders and non-responders, there is a difference in the social circumstances as responders had higher WIMD scores. We did account for this in our analysis and it should be noted the response rate for preterm born children was 32%.

## Conclusion

This large population-based study has confirmed and further quantified that preterm-born children have increased prevalence of respiratory symptoms. Children born preterm with or without a family history of atopy had a higher prevalence of wheezing and were more frequently on inhaler treatment when compared to term-born children. Prematurity-associated wheeze is likely to be a separate entity to the wheezing observed in term-born children especially as delivery occurs at an earlier stage of lung growth, which may affect subsequent lung development. It is vital that we identify optimal treatment for this significant group of children by identifying the underlying mechanisms responsible for the airway symptoms and obstruction together with adequately powered randomised controlled trials.

## Supporting Information

S1 File **Table A**: Characteristics of non-responders <5 years of age according to gestational groups. **Table B**: Characteristics of non-responders ≥5 years of age according to gestational groups. **Table C**: Univariate analysis of risk factors for preterm wheezing children less than 5 years-of-age (unadjusted OR). **Table D**: Univariate analysis of risk factors for preterm wheezing children 5 years-of-age and older (unadjusted OR). **Table E**: Characteristics of very preterm born children with and without CLD compared to full term born controls. **Table F**: Characteristics of matched case-control responders <5 years of age according to gestational groups. **Table G**: Characteristics of matched case-control responders’ ≥5 years of age according to gestational groups. **Table H**: Childhood wheezing for all children less than 5 years-of-age compared by gestational age for matched case-controls only (unadjusted OR). **Table I**: Childhood wheezing for all children 5 years-of-age and older compared by gestational age for matched case-controls only (unadjusted OR).
(DOCX)
