# Supplementary material for: Management of Prematurity-Associated Wheeze and Its Association with Atopy
Source: PLoS One. 2016 May 20;11(5):e0155695. doi: 10.1371/journal.pone.0155695 (PMC4874578; doi:10.1371/journal.pone.0155695)
Supplement: S1 File — Table A: Characteristics of non-responders <5 years of age according to gestational groups. Table B: Characteristics of non-responders ≥5 years of age according to gestational groups. Table C: Univariate analysis of risk factors for preterm wheezing children less than 5 years-of-age (unadjusted OR). Table D: Univariate analysis of risk factors for preterm wheezing children 5 years-of-age and older (unadjusted OR). Table E: Characteristics of very preterm born children with and without CLD compared to full term born controls. Table F: Characteristics of matched case-control responders <5 years of age according to gestational groups. Table G: Characteristics of matched case-control responders’ ≥5 years of age according to gestational groups. Table H: Childhood wheezing for all children less than 5 years-of-age compared by gestational age for matched case-controls only (unadjusted OR). Table I: Childhood wheezing for all children 5 years-of-age and older compared by gestational age for matched case-controls only (unadjusted OR). (DOCX) [file pone.0155695.s001.docx]

**Cover Sheet: online data supplement**

**Management of prematurity-associated wheeze and its association with atopy**

^1^Martin O Edwards, ^1^Sarah J Kotecha, ^1^John Lowe, ^2^Louise Richards, ^1^W John Watkins, ^1^Sailesh Kotecha.

**Table A: Characteristics of non-responders <5 years of age according to gestational groups**

|  | **Very Preterm**  **N =905** | **Moderate Preterm**  **N=1006** | **Late Preterm**  **N=2894** | **Full Term**  **N=5554** |
| --- | --- | --- | --- | --- |
| **Gestational age, weeks**  **(mean, 95%CI)** | 29.68  (29.53, 29.82) | 33.63  (33.60, 33.66) | 35.65  (35.63, 35.67) | 39.70  (39.66, 39.73)*  p = 0.028 |
| **Birth weight, kg**  **(mean, 95%CI)** | 1.49  (1.46, 1.53) | 2.176  (2.148, 2.205) | 2.618  (2.601, 2.636) | 3.449  (3.436, 3.462) |
| **Male (%)** | 501 (55.4%)  p = 0.055 | 560 (55.7%)  p>>0.05 | 1555 (53.7%)  p>>0.05 | 3027 (54.5%)  p>>0.05 |
| **WIMD rank latest**  **(median)** | 617*  p≤0.001 | 644*  p≤0.001 | 639*  p≤0.001 | 745*  p≤0.001 |
| **Age, years (mean, 95%CI)** | 2.96 (2.91, 3.01)*  p≤0.001 | 2.99 (2.94, 3.04)*  p≤0.001 | 2.98 (2.95, 3.01)*  p≤0.001 | 2.99 (2.97, 3.01)*  p≤0.001 |

**Significance levels relates to comparison of characteristics of responders.*

**Table B: Characteristics of non-responders ≥5 years of age according to gestational groups**

|  | **Very Preterm**  **N =833** | **Moderate Preterm**  **N=956** | **Late Preterm**  **N=2496** | **Full Term**  **N=4949** |
| --- | --- | --- | --- | --- |
| **Gestational age, weeks**  **(mean, 95%CI)** | 29.68  (29.53, 29.83) | 33.61  (33.58, 33.64) | 35.64  (35.62, 35.65) | 39.67  (39.64, 39.71) |
| **Birth weight, kg**  **(mean, 95%CI)** | 1.515  (1.477, 1.553)*  p=0.023 | 2.192  (2.160, 2.223) | 2.624  (2.605, 2.643) | 3.434  (3.420, 3.448)  p=0.077 |
| **Male (%)** | 448 (53.8%)  p=0.055 | 519 (54.3%)  p>>0.05 | 1340 (53.7%)  p>>0.05 | 2716 (54.9%)  p>>0.05 |
| **WIMD rank latest**  **(median)** | 641*  p≤0.001 | 710*  p≤0.001 | 723*  p≤0.001 | 810*  p≤0.001 |
| **Age, years (mean, 95%CI)** | 7.90 (7.79, 8.01)*  p≤0.001 | 7.87 (7.76, 7.97)*  p≤0.001 | 7.90 (7.83, 7.96)*  p≤0.001 | 7.90 (7.85, 7.94)*  p≤0.001 |

**Significance levels relates to comparison of characteristics of responders.*

**Table C: Univariate analysis of risk factors for preterm wheezing children less than 5 years-of-age (unadjusted OR)**

| **Wheezing ever (<5 years old)** | **Preterm wheezers** | **Term wheezers** | **OR**  **(95% CI)** |
| --- | --- | --- | --- |
| **Family history of Atopy +**  **-** | 409/1172 (34.9%)  763/1172 (65.1%) | 192/569 (33.8%)  377/569 (66.2%) | 1.1 (0.9, 1.3)  P=0.64 |
| **Maternal smoking during YES**  **pregnancy NO** | 156/1172 (13.3%)  1016/1172 (86.7%) | 63/569 (11.1%)  506/569 (88.9%) | 1.2 (0.9, 1.7)  p=0.19 |
| **Mode of delivery CS**  **Non-CS** | 579/1103 (52.5%)  524/1103 (47.5%) | 164/549 (29.9%)  385/549 (70.1%) | **2.6 (2.1, 3.2)**  **p <0.001** |
| **IUGR <10^th^ centile**  **20^th^-80^th^ centile** | 137/792 (17.3%)  655/792 (82.7%) | 36/396 (9.1%)  360/396 (90.9%) | **2.1 (1.4, 3.1)**  **p <0.001** |
| **Gender Male**  **Female** | 666/1172 (56.8%)  506/1172 (43.2%) | 335/569 (58.8%)  234/569 (41.1%) | 0.9 (0.8, 1.1)  p=0.42 |
| **Current maternal smoking YES**  **NO** | 227/1172 (19.4%)  945/1172 (80.6%) | 81/571 (14.2%)  490/571 (85.8%) | **1.5 (1.1, 1.9)**  **P= 0.008** |

**Table D: Univariate analysis of risk factors for preterm wheezing children 5 years-of-age and older (unadjusted OR)**

| **Wheezing ever (≥5 years old)** | **Preterm wheezers** | **Term wheezers** | **OR**  **(95% CI)** |
| --- | --- | --- | --- |
| **Family history of Atopy +**  **-** | 259/892 (29%)  633/892 (71%) | 132/402 (32.8%)  270/402 (67.2%) | 0.8 (0.7, 1.1)  p=0.17 |
| **Maternal smoking during YES**  **pregnancy NO** | 143/892 (16%)  749/892 (84%) | 55/402 (13.7%)  347/402 (86.3%) | 1.2 (0.9, 1.7)  p=0.28 |
| **Mode of delivery CS**  **Non-CS** | 399/812 (49.1%)  413/812 (50.9%) | 102/354 (28.8%)  252/354 (71.2%) | **2.4 (1.8, 3.1)**  **p<0.001** |
| **IUGR <10^th^ centile**  **20^th^-80^th^ centile** | 104/604 (17.2%)  500/604 (82.8%) | 32/296 (10.8%)  264/296 (89.2%) | **1.7 (1.1, 2.6)**  **p=0.012** |
| **Gender Male**  **Female** | 545/892 (61.1%)  347/892 (38.9%) | 237/402 (59.0%)  165/402 (41.0%) | 1.1 (0.9, 1.4)  p=0.47 |
| **Current maternal Yes**  **smoking No** | 181/892 (20.3%)  711/892 (79.7%) | 64/402 (15.9%)  338/402 (84.1%) | 1.3 (0.99, 1.8)  p=0.064 |

**Table E: Characteristics of very preterm born children with and without CLD compared to full term born controls.**

|  | **CLD**  **N=152** | **No CLD**  **N= 845** | **Full Term**  **N=2858** |
| --- | --- | --- | --- |
| **Gestational age, weeks**  **(mean, 95%CI)** | 27.11  (26.8, 27.5) | 30.2  (30.1, 30.3) | 39.6  (39.6, 39.7) |
| **Birth weight, kg**  **(mean, 95%CI)** | 0.98 (0.93, 1.03) | 1.48 (1.46, 1.51) | 3.46  (3.44, 3.48) |
| **Male (%)** | 87 (57%) | 457 (54%) | 1513 (53%) |
| **WIMD rank latest**  **(median)** | 927 | 967 | 1056 |
| **Age, years**  **(mean, 95% CI)** | 4.2 (3.78, 4.66) | 4.8 (4.57, 4.95) | 4.8 (4.67, 4.89)  (7.1, 7.3) |
| **Mother smoke (in pregnancy)** | 24 (15.8%) | 116 (13.7%) | 317 (11.1%) |
| **Maternal age, years**  **(mean, 95%CI)** | 29.0 (27.9, 30.1) | 30.3 (29.9, 30.8) | 30.4 (30.1, 30.6) |

**Table F: Characteristics of matched case-control responders <5 years of age according to gestational groups**

|  | **Very Preterm**  **N = 125** | **Moderate Preterm**  **N=141** | **Late Preterm**  **N=362** | **Full Term**  **N=628** |
| --- | --- | --- | --- | --- |
| **Gestational age, weeks**  **(mean, 95%CI)** | 29.7*  (29.4,30.0) | 33.7*  (33.6,33.8) | 35.7*  (35.6,35.7) | 39.6*  (39.5,39.7) |
| **Birthweight, kg**  **(mean, 95%CI)** | 1.46*  (1.37, 1.56) | 2.20*  (2.13, 2.29) | 2.64*  (2.59, 2.68) | 3.48*  (3.44, 3.51) |
| **Male (%)** | 64 (51%) | 82 (58%) | 219 (61%) | 365 (58%) |
| **WIMD rank (median)**  **Range: 1-1909** | 1018 | 947 | 916 | 949 |
| **Age, years**  **(mean, 95% CI)** | 2.32  (2.16, 2.48) | 2.26  (2.12, 2.40) | 2.28  (2.18, 2.37) | 2.29  (2.22, 2.36) |
| **Antenatal maternal smoking** | 17/124  (13.7%) | 22/141  (15.6%) | 62/299  (17.2%) | 73/553  (11.7%) |
| **Maternal age, years**  **(mean, 95%CI)** | 30.3  (29.1, 31.4) | 30.9  (29.8, 31.9) | 30.1  (29.5, 30.8) | 30.5  (30.0, 31.0) |
| **Mode of delivery (CS or not) Missing data** | 66 (56.4%)*  8 (6.4%) | 78 (56.1%)*  2 (1.4%) | 159 (45.2%)*  10 (2.8%) | 179 (29.1%)*  12 (1.9%) |
| **IUGR** | 16 (12.8%)* | 17 (12.1%)* | 34 (9.4%)* | 39(6.2%)* |

*****Significant difference (p<0.001) between groups.

**Table G: Characteristics of matched case-control responders’ ≥5 years of age according to gestational groups**

|  | **Very Preterm**  **N =162** | **Moderate Preterm**  **N=135** | **Late Preterm**  **N=335** | **Full Term**  **N=632** |
| --- | --- | --- | --- | --- |
| **Gestational age, weeks**  **(mean, 95%CI)** | 29.8*  (29.4, 30.1) | 33.6*  (33.5, 33.7) | 35.6*  (35.6, 35.7) | 39.6*  (39.6, 39.7) |
| **Birthweight, kg**  **(mean, 95%CI)** | 1.45*  (1.38, 1.52) | 2.20*  (2.12, 2.28) | 2.63*  (2.58, 2.68) | 3.48*  (3.44, 3.52) |
| **Male (%)** | 96 (59.3%) | 65 (48.1%) | 200 (59.7%) | 361 (57.1%) |
| **WIMD rank (median)**  **Range: 1-1909** | 836* | 880* | 1,038* | 1,150* |
| **Age, years (mean, 95%CI)** | 7.0  (6.8, 7.3) | 7.2  (6.9, 7.5) | 7.2  (7.0, 7.4) | 7.2  (7.1, 7.3) |
| **Antenatal maternal smoking** | 32/129  (19.9%) | 28/106  (20.9%) | 55/280  (16.4%) | 91/539  (14.4%) |
| **Maternal age, years**  **(mean, 95%CI)** | 30.2  (29.1, 31.2) | 31.1  (30.0, 32.1) | 30.1  (29.4, 30.8) | 30.3  (29.9, 30.8) |
| **Mode of delivery (CS or not) Missing data** | 92 (62.5%)*  10 (6.2%) | 73 (57%)*  7 (5.2%) | 130 (40.4%)*  3 (0.9%) | 162 (28.5%)*  63 (10%) |
| **IUGR** | 23 (14.2%)* | 13 (9.6%)* | 34 (9.4%)* | 39 (6.2%)* |

*****Significant difference (p<0.001) between groups.

**Table H: Childhood wheezing for all children less than 5 years-of-age compared by gestational age for matched case-controls only (unadjusted OR)**

|  | **Very Preterm**  **N =125** | **Moderate Preterm**  **N=141** | **Late Preterm**  **N=362** | **Full Term**  **N=628** |
| --- | --- | --- | --- | --- |
| **Wheeze-ever (%)**  **OR (95% CI)**  **p-value** | 81 (64.8%)  2.4 (1.6, 3.5)  <0.001 | 74 (52.5%)  1.4 (0.98, 2.0)  0.06 | 203 (56.1%)  1.6 (1.3, 2.1)  <0.001 | 275 (43.8%) |
| **Recent wheeze (%)**  **OR (95% CI)**  **p-value** | 50 (40%)  2.7 (1.8, 4.0)  <0.001 | 42 (29.8%)  1.7 (1.1, 2.6)  <0.001 | 107 (29.6%)  1.7 (1.2, 2.3)  <0.001 | 126 (20.1%) |

**Table I: Childhood wheezing for all children 5 years-of-age and older compared by gestational age for matched case-controls only (unadjusted OR)**

|  | **Very Preterm**  **N = 162** | **Moderate Preterm**  **N= 135** | **Late Preterm**  **N= 335** | **Full Term**  **N= 632** |
| --- | --- | --- | --- | --- |
| **Wheeze-ever (%)**  **OR (95% CI)**  **p-value** | 80 (49.4%)  2.7 (1.9, 3.8)  <0.001 | 56 (41.5%)  2.0 (1.3, 2.9)  <0.001 | 132 (39.4%)  1.8 (1.4, 2.4)  <0.001 | 168 (26.6%) |
| **Recent wheeze (%)**  **OR (95% CI)**  **p-value** | 48 (29.6%)  2.7 (1.8, 4.1)  <0.001 | 33 (24.4%)  2.1 (1.3, 3.3)  <0.001 | 65 (19.4%)  1.6 (1.1, 2.2)  0.001 | 85 (13.4%) |
